# Compressive Force During Orthodontic Tooth Movement Triggers Ferroptosis and Suppresses Osteogenic Differentiation of Human Periodontal Ligament Stem Cells Through SCD1 Downregulation

**DOI:** 10.3390/antiox15101292

**Published:** 2026-10-05

**Authors:** Tianjing Du, Jiamin Zhao, Xin Shao, Minyue Zhao, Wenhao Weng, Ruibo Zhu, Zhihui Feng, Zhongbo Liu, Kun Qi

**Affiliations:** 1 Key Laboratory of Shaanxi Province for Craniofacial Precision Medicine Research, College of Stomatology, Xi’an Jiaotong University, Xi’an 710004, China; karma0220@stu.xjtu.edu.cn (T.D.); zhaojiamin@xjtu.edu.cn (J.Z.); shaoxin1128@stu.xjtu.edu.cn (X.S.); minyue1212@stu.xjtu.edu.cn (M.Z.); wengwenhao@stu.xjtu.edu.cn (W.W.); zrb_bo@stu.xjtu.edu.cn (R.Z.); 2 Laboratory Center of Stomatology, College of Stomatology, Xi’an Jiaotong University, Xi’an 710004, China; 3 Department of Orthodontics, College of Stomatology, Xi’an Jiaotong University, Xi’an 710004, China; 4 Frontier Institute of Science and Technology, Xi’an Jiaotong University, Xi’an 710049, China; zhfeng@xjtu.edu.cn; 5 Interdisciplinary Research Center of Frontier Science and Technology, Xi’an Jiaotong University, Xi’an 710049, China

**Keywords:** ferroptosis, lipid peroxidation, stearoyl-CoA desaturase 1, human periodontal ligament stem cells, compressive force, osteogenic differentiation, orthodontic tooth movement

## Abstract

Improper orthodontic treatment can disrupt alveolar bone remodeling, leading to excessive alveolar bone resorption and complications such as bone fenestration and dehiscence. Although ferroptosis has emerged as an important regulator of bone metabolism, its role in mechanical force-induced periodontal tissue remodeling during orthodontic tooth movement (OTM) remains incompletely understood. This study investigated whether ferroptosis regulates the osteogenic response of human periodontal ligament stem cells (hPDLSCs) under compressive force (CF), focusing on stearoyl-CoA desaturase 1 (SCD1). hPDLSCs were subjected to static CF, and RNA sequencing, RT–qPCR, Western blotting, and immunofluorescence were performed to analyze SREBP1 and SCD1 expression. Ferroptosis-related alterations were evaluated using biochemical and mitochondrial analyses. Ferrostatin-1 (Fer-1) rescue, SCD1 knockdown/overexpression, and a rat OTM model were used for functional validation. Prolonged CF downregulated SREBP1 and SCD1 expression, accompanied by ferroptosis-associated alterations and reduced RUNX2 and OPN expression. Fer-1 alleviated these changes, whereas SCD1 overexpression partially restored osteogenic markers and SCD1 knockdown further impaired osteogenic responses. In vivo, SREBP1 and SCD1 expression were reduced on the compression side. Collectively, these findings identify SCD1 as an important regulator linking CF-induced ferroptosis-associated alterations with impaired osteogenic differentiation of hPDLSCs, suggesting SCD1 as a potential target for orthodontic bone remodeling.

## 1. Introduction

Malocclusion represents one of the most prevalent oral health disorders worldwide, with a reported global prevalence of approximately 56% across different stages of dentition [1]. Orthodontic treatment, through the application of controlled mechanical forces, remains the primary therapeutic strategy for correcting malocclusion and improving occlusal function and facial aesthetics [2]. The biological foundation of orthodontic treatment is orthodontic tooth movement (OTM), a process that relies on spatiotemporally regulated alveolar bone remodeling in response to mechanical loading [3,4]. According to the widely accepted pressure–tension theory [5], orthodontic force generates two distinct tissue compartments: the tension side, where osteoblast-mediated bone formation predominates, and the compression side, where osteoclast-driven bone resorption occurs [6]. Human periodontal ligament stem cells (hPDLSCs), as mechanosensitive progenitor cells residing within periodontal ligament tissues, play a pivotal role in orthodontic force-induced alveolar bone remodeling through their multipotent osteogenic differentiation capacity and paracrine regulation of the local periodontal microenvironment [7]. However, when the delicate balance between bone formation and resorption is disrupted during orthodontic treatment, pathological complications may arise, including excessive alveolar bone resorption, bone fenestration, and bone dehiscence, all of which compromise long-term periodontal support, treatment stability, and clinical outcomes [8,9,10].

Mechanical forces applied during orthodontic treatment can induce dynamic changes in cellular metabolism and redox homeostasis within periodontal tissues. Such metabolic disturbances may influence cell fate decisions and contribute to abnormal alveolar bone remodeling [11]. Ferroptosis, a recently identified form of regulated cell death distinct from apoptosis and necrosis [12,13,14,15], has emerged as a critical player in various bone-related pathologies [16,17,18]. It is characterized by iron-dependent accumulation of lipid peroxides and distinctive mitochondrial morphological changes—including shrinkage, increased membrane density, and cristae reduction—while nuclear integrity is preserved [19]. Accumulating evidence indicates that ferroptosis is intimately linked to metabolic dysregulation in bone homeostasis: excess intracellular iron catalyzes Fenton reactions, generating excessive reactive oxygen species (ROS) that drive lipid peroxidation and impair osteoblast/osteocyte viability, ultimately tipping the balance toward bone resorption [20]. Moreover, elevated ROS levels can upregulate receptor activator of nuclear factor-κB ligand (RANKL) expression, increase the RANKL/osteoprotegerin (OPG) ratio, and promote osteoclast differentiation and bone resorption [21], collectively disrupting bone homeostasis and accelerating pathological bone loss [22]. Given that orthodontic compressive force (CF) is well documented to induce oxidative stress in periodontal tissues, ferroptosis may represent a previously unrecognized mechanism underlying force-induced alveolar bone remodeling imbalance.

Lipid metabolism is increasingly recognized as a central regulator of ferroptosis susceptibility by modulating the availability and composition of peroxidation-prone membrane lipids [23,24]. Stearoyl-CoA desaturase 1 (SCD1; encoded by *SCD*) is an endoplasmic reticulum-resident Δ9 fatty acid desaturase that catalyzes the rate-limiting conversion of saturated fatty acids (SFAs), primarily palmitic acid and stearic acid, into the monounsaturated fatty acids (MUFAs) palmitoleic acid and oleic acid. By increasing MUFA production and reducing the incorporation of oxidation-prone polyunsaturated fatty acids (PUFAs) into membrane phospholipids, SCD1 limits lipid peroxidation and protects cells against ferroptotic damage [25]. Upstream of SCD1, sterol regulatory element-binding protein 1 (SREBP1; encoded by *SREBF1*) is a master transcription factor that orchestrates the expression of genes involved in de novo lipid synthesis and fatty acid uptake, including direct transcriptional regulation of SCD1. Consequently, SREBP1-mediated lipid metabolic programs are considered important determinants of ferroptosis susceptibility by regulating membrane lipid composition [26]. Importantly, SREBP1 has been identified as a mechanosensitive metabolic regulator that couples extracellular mechanical cues with lipid metabolic reprogramming and cell fate decisions [27]. As documented in multiple cell types, SREBP1 is a well-characterized upstream transcription factor that directly regulates SCD1 expression and governs global lipogenic programs [27,28]. Although this SREBP1–SCD1 regulatory relationship has been extensively validated in the previous literature, whether alterations in SCD1-mediated lipid metabolism contribute to mechanical force-induced ferroptosis in hPDLSCs remains unexplored. The present study mainly focuses on the functional role of SCD1 in force-induced ferroptosis and osteogenic regulation, with SREBP1 detected as an indicator of the overall lipogenic status under compressive loading.

Emerging evidence has begun to implicate ferroptosis in alveolar bone remodeling during OTM. For instance, CF was shown to downregulate glutathione peroxidase 4 (GPX4) and induce ferroptosis in MC3T3-E1 preosteoblasts, thereby suppressing osteogenic activity via the YAP/TAZ signaling pathway [29]. In addition, prostaglandin E2 (PGE2) was recently reported to modulate ferroptosis and osteogenesis in MC3T3-E1 cells through nitric oxide synthase 2 (NOS2) regulation [30]. However, current research remains limited in several critical aspects: first, most studies have focused on osteoblastic cell lines rather than hPDLSCs—the primary mechanosensitive cells in periodontal tissues that directly mediate OTM [31,32]; second, the specific role of SCD1-mediated lipid metabolic dysregulation in force-induced ferroptosis remains entirely unexplored; and third, whether ferroptosis directly contributes to the impaired osteogenic capacity of hPDLSCs under CF has not been established.

Therefore, we hypothesize that CF induces ferroptosis in hPDLSCs through downregulating SCD1-mediated lipid metabolism, which in turn suppresses osteogenic differentiation and contributes to alveolar bone remodeling imbalance during OTM. SREBP1, as a known upstream transcriptional regulator of SCD1, may also be involved in this process. This study aims to (1) characterize ferroptotic phenotypes in hPDLSCs under CF, (2) determine the functional role of SCD1 in force-induced ferroptosis and osteogenic suppression, and (3) validate the in vivo relevance of these findings using a rat OTM model. Our findings may provide novel mechanistic insights into orthodontic alveolar bone remodeling and identify potential antioxidant therapeutic targets for preventing excessive bone resorption during orthodontic treatment.

## 2. Materials and Methods

### 2.1. Animals and Orthodontic Force Application

All animal experiments were approved by the Biomedical Ethics Committee of the Health Science Center, Xi’an Jiaotong University (approval No. XJTUAE2024-16). Twenty male Sprague–Dawley rats (8 weeks old; body weight 200 ± 20 g) were purchased from the Laboratory Animal Center of Xi’an Jiaotong University. After 1 week of acclimatization, the rats were randomly assigned to four groups (*n* = 5 per group): control group, OTM 3-day group, OTM 7-day group, and OTM 14-day group. All animals were housed under specific pathogen-free (SPF) conditions with a 12 h light/dark cycle and free access to standard chow and water.

After anesthesia via intraperitoneal injection of 1% sodium pentobarbital (4 mL/kg body weight), orthodontic appliances were placed as previously described by King et al. [33]. The OTM model was established using a nickel–titanium closed-coil spring. A 5 cm segment of 0.2 mm orthodontic ligature wire was passed through the buccal interdental space between the right maxillary first and second molars, looped around the cervical region of the first molar, and tightly ligated to secure the posterior end of the coil spring to the mesial aspect of the first molar. The spring was then stretched while the applied force was monitored using a spring force gauge. When the force reached approximately 50 g, the corresponding activation position of the spring was marked. The maxillary incisors were etched, and another 5 cm segment of 0.2 mm orthodontic ligature wire was passed through the anterior end of the coil spring and ligated around both maxillary incisors as anchorage, with the spring maintained at the previously marked position to generate an initial orthodontic force of approximately 50 g. Adhesive and flowable resin were subsequently applied to the maxillary incisors to reinforce the ligature and stabilize the appliance. Consistent with previously reported rat OTM studies [34,35,36,37], rats in the OTM groups were euthanized with an overdose of sodium pentobarbital on days 3, 7, and 14 after appliance placement, respectively, and maxillary tissue blocks containing the first molar were harvested for subsequent analyses.

### 2.2. Micro-Computed Tomography (Micro-CT) Analysis

Maxillary samples were fixed in 4% paraformaldehyde and scanned using a high-resolution micro-CT system (Quantum GX2, PerkinElmer, Waltham, MA, USA) with the following parameters: 90 kV, 88 μA, and 20 μm isotropic voxel size. The acquired DICOM datasets were imported into Mimics 21 software (Materialise, Leuven, Belgium) for three-dimensional reconstruction and linear measurement. The OTM distance was defined as the linear distance between the midpoints of the distal marginal ridge of the right maxillary first molar and the mesial marginal ridge of the adjacent second molar. All measurements were performed in triplicate by two independent observers blinded to group allocation, and the mean value was used for statistical analysis.

### 2.3. Hematoxylin and Eosin (H&E) and Tartrate-Resistant Acid Phosphatase (TRAP) Staining

Maxillary samples were fixed in 4% paraformaldehyde for 48 h, decalcified in 10% EDTA (pH 7.4) for 12 weeks at room temperature with weekly solution changes, dehydrated through a graded ethanol series, and embedded in paraffin. Serial sagittal sections (5 μm thickness) were prepared using a microtome (Leica, Wetzlar, Germany).

H&E staining was performed according to standard protocols to examine periodontal tissue morphology on both the compression and tension sides. TRAP staining was conducted on sections from the compression side using a commercial TRAP staining kit (Solarbio, Beijing, China) following the manufacturer’s instructions. TRAP-positive multinucleated osteoclasts (containing ≥3 nuclei) were identified under light microscopy within predefined regions of interest (ROIs) in the periodontal ligament region on the compression side of the right maxillary first molar. The number of TRAP^+^ cells was quantified and expressed as the mean number of TRAP-positive cells per mm^2^ of periodontal ligament area. Quantification was performed by two independent observers blinded to group allocation using ImageJ v1.53t software (National Institutes of Health, Bethesda, MD, USA).

### 2.4. Immunohistochemical (IHC) Staining

IHC staining was performed on paraffin sections from the compression side to assess the protein expression levels of SCD1 and SREBP1. Briefly, sections were deparaffinized in xylene, rehydrated through a graded ethanol series, and outlined with a hydrophobic barrier pen (Zhongshan Golden Bridge, Beijing, China). Endogenous peroxidase activity was quenched with 3% hydrogen peroxide (Boster, Wuhan, China) for 10 min at room temperature. Antigen retrieval was performed using compound digestive solution (Boster, Wuhan, China) at 37 °C for 30 min. After blocking with 5% bovine serum albumin (BSA; Boster, Wuhan, China) for 30 min at room temperature, sections were incubated overnight at 4 °C with primary antibodies against SCD1 (1:300, 28678-1-AP; Proteintech, Wuhan, China) and SREBP1 (1:100, 14088-1-AP; Proteintech). Negative control sections were incubated with PBS instead of primary antibody.

After three washes with PBS, sections were incubated with horseradish peroxidase (HRP)-conjugated goat anti-rabbit IgG secondary antibody (Boster, Wuhan, China) for 30 min at 37 °C, followed by incubation with streptavidin-biotin complex (SABC; Boster, Wuhan, China) for 30 min at 37 °C. Immunoreactivity was visualized using 3,3’-diaminobenzidine (DAB) chromogen (Boster, Wuhan, China), and the reaction was stopped by immersion in distilled water. Sections were then counterstained with hematoxylin, differentiated in acid alcohol, blued in Scott’s tap water substitute, dehydrated, cleared, and mounted with neutral balsam.

For each sample, five non-overlapping fields from the compression side periodontal ligament region were randomly selected and analyzed. Immunopositive signals were quantified using Image-Pro Plus v6.0 software (Media Cybernetics, Rockville, MD, USA) by measuring the integrated optical density (IOD) and the percentage of positive staining area. All evaluations were performed by two independent observers blinded to group allocation.

### 2.5. Cell Isolation, Culture, and Treatments

This study was approved by the Ethics Committee of the Stomatology Hospital of Xi’an Jiaotong University (approval No. KY-GXB-20230012), and written informed consent was obtained from all participating patients. hPDLSCs were isolated from caries-free, periodontally healthy premolars extracted for orthodontic purposes from patients aged 16–25 years. Briefly, after rinsing with sterile PBS, periodontal ligament tissue from the middle third of the root surface was gently scraped and minced into small pieces. The tissue fragments were digested with 3 mg/mL type I collagenase (Thermo Fisher Scientific, Waltham, MA, USA) and 4 mg/mL neutral protease (Roche Diagnostics GmbH, Mannheim, Germany) at 37 °C for 40–50 min with gentle agitation. The cell suspension was filtered through a 70 μm cell strainer and plated in α-minimum essential medium (α-MEM; Gibco, Waltham, MA, USA) supplemented with 20% fetal bovine serum (FBS; Hangzhou Sijiqing Biological Engineering Materials Co., Ltd., Hangzhou, Zhejiang, China) and 1% penicillin/streptomycin. Cells were maintained at 37 °C in a humidified atmosphere with 5% CO_2_. Cells at passages 3–5 were used for experiments.

For ferrostatin-1 (Fer-1) rescue experiments, Fer-1 (cat. No. HY-100579, MCE, Monmouth Junction, NJ, USA) was dissolved in dimethyl sulfoxide (DMSO) to prepare a 10 mM stock solution, which was aliquoted, protected from light, and stored at −20 °C. Immediately before use, the stock solution was diluted in complete culture medium to the working concentration (5 μM), with a final DMSO concentration ≤ 0.1%. hPDLSCs were preincubated with Fer-1 for 1 h prior to the application of CF.

### 2.6. Application of CF

hPDLSCs were seeded in 6-well plates at a density of 1 × 10^5^ cells/well and subjected to compressive loading when they reached 80–90% confluence. As previously described by Kanzaki et al. [38], a 30 mm sterile glass coverslip was placed over the cell monolayer, and a glass cylinder containing lead granules was positioned on the coverslip to apply a static compressive load. The CF was determined according to the applied load and the contact area of the coverslip, and the amount of lead granules was adjusted to achieve a force of 2.0 g/cm^2^. Cells were continuously subjected to CF for 24 or 48 h. Control cells were covered with the same sterile glass coverslip but without the additional loading apparatus.

### 2.7. Cell Differentiation Assays

The osteogenic and adipogenic differentiation potential of hPDLSCs was assessed using standard induction protocols as previously reported [39].

For osteogenic differentiation, when cells reached ~80% confluence, the culture medium was replaced with osteogenic induction medium consisting of α-MEM supplemented with 10% FBS and the following additives: 0.05 mM L-ascorbic acid 2-phosphate, 10 mM β-glycerophosphate, and 100 nM dexamethasone (all from Sigma-Aldrich, St. Louis, MO, USA). The induction medium was replaced every 2 days. Alkaline phosphatase (ALP) staining was performed on day 7 using a BCIP/NBT ALP staining kit (Beyotime, Shanghai, China) according to the manufacturer’s instructions. Alizarin Red S (ARS) staining (1%, Solarbio, Beijing, China) was performed on day 21 to evaluate extracellular matrix mineralization and calcium nodule formation.

For adipogenic differentiation, cells at ~80% confluence were induced using a commercial adipogenic differentiation kit (Cyagen, Santa Clara, CA, USA) according to the manufacturer’s protocol. Briefly, cells were cultured alternately with Induction Solution A for 3 days and Maintenance Solution B for 1 day for a total of 14 days. Oil Red O staining (Cyagen, Santa Clara, CA, USA) was performed to visualize intracellular lipid droplet accumulation.

### 2.8. Flow Cytometry

Flow cytometry was used to assess the immunophenotypic surface marker expression of hPDLSCs. Single-cell suspensions were prepared and diluted in PBS containing 3% FBS to a concentration of 1 × 10^6^ cells/mL. Aliquots of 300 μL cell suspension were dispensed into 1.5 mL microcentrifuge tubes. Cells were incubated on ice for 30 min in the dark with the following fluorochrome-conjugated antibodies against STRO-1 (FITC), CD105 (PE), CD90 (FITC), CD34 (PE), and CD45 (PE) (all from R&D Systems, Minneapolis, MN, USA). An unstained cell suspension was included as a negative control.

After staining, cells were washed twice with cold PBS and centrifuged at 300× *g* for 5 min at 4 °C. The supernatant was discarded, and cell pellets were resuspended in 300 μL PBS for immediate flow cytometric acquisition. Data were analyzed using FlowJo™ v10.0 Software (BD Life Sciences, Franklin Lakes, OR, USA).

### 2.9. RNA Sequencing and Bioinformatics Analysis

hPDLSCs were subjected to a CF of 2.0 g/cm^2^ for 24 h, and unloaded cells served as controls. Each group comprised three independent biological replicates (*n* = 3). Total RNA was extracted using TRIzol reagent (Sparkjade, Jinan, Shandong, China) according to the manufacturer’s instructions. RNA quantity and integrity were assessed using a NanoDrop ND-1000 spectrophotometer (Thermo Fisher Scientific, Waltham, MA, USA) and an Agilent 2100 Bioanalyzer (Agilent Technologies, Santa Clara, CA, USA). Samples meeting the following quality criteria were used for sequencing: RNA concentration > 50 ng/μL, RNA integrity number (RIN) > 7.0, OD_260/280_ > 1.8, and total RNA > 1 μg.

cDNA libraries were prepared and sequenced on the Illumina NovaSeq™ 6000 platform (LC-Bio Technology Co., Ltd., Hangzhou, China) to generate paired-end 150 bp reads. Raw reads were quality-filtered using fastp v0.23.2 software, and clean reads were aligned to the human reference genome using HISAT2. Transcript assembly and quantification were performed with StringTie, and gene expression levels were normalized and reported as fragments per kilobase of transcript per million mapped reads (FPKM).

Differentially expressed genes (DEGs) between the compression and control groups were identified using the edgeR package with a threshold of *q*-value < 0.05 and an absolute fold change ≥ 1.2. Ferroptosis-related genes (FRGs) were retrieved from the FerrDb database. Venn analysis was performed to identify the overlap between DEGs and FRGs, yielding ferroptosis-related differentially expressed genes (FRDEGs). Functional enrichment analyses, including Gene Ontology (GO) and Kyoto Encyclopedia of Genes and Genomes (KEGG) analyses, were performed using the DAVID database. Protein–protein interaction (PPI) networks were constructed using the STRING database, and hub genes were ranked by connectivity degree using the cytoHubba plugin in Cytoscape v3.10 (Cytoscape Consortium, San Diego, CA, USA).

### 2.10. Intracellular ROS Assay

Intracellular ROS levels were assessed using the 2’,7’-dichlorodihydrofluorescein diacetate (DCFH-DA) fluorescent probe (Beyotime, Shanghai, China). Briefly, after the indicated treatments, hPDLSCs were incubated with 10 μM DCFH-DA working solution at 37 °C for 20 min in the dark. Cells were then washed twice with serum-free medium to remove extracellular probe. Fluorescence images were acquired using an FV3000 confocal laser scanning microscope (Olympus, Tokyo, Japan), and the mean fluorescence intensity was quantified using ImageJ v1.53t software.

### 2.11. Lipid ROS Assay

Lipid ROS levels were evaluated using the C11-BODIPY 581/591 probe (Thermo Fisher Scientific, Waltham, MA, USA), which specifically detects lipid peroxidation in cellular membranes. After the indicated treatments, hPDLSCs were incubated with 5 μM C11-BODIPY 581/591 working solution at 37 °C for 30 min in the dark, followed by two washes with PBS. Fluorescence images were acquired using an FV3000 confocal microscope, and the fluorescence intensity was calculated to quantify lipid ROS levels using ImageJ v1.53t software.

### 2.12. Mitochondrial Membrane Potential Measurement

Mitochondrial membrane potential (Δψm) was measured using a JC-1 probe kit (Beyotime, Shanghai, China) according to the manufacturer’s instructions. The JC-1 working solution was prepared by mixing 50 μL of JC-1 dye (200×) with 2 mL of staining buffer and 8 mL of ultrapure water. After the indicated treatments, cells were incubated with the JC-1 working solution at 37 °C for 20 min in the dark and then washed twice with JC-1 buffer. Fluorescence images were acquired using an FV3000 confocal microscope. Δψm was evaluated based on the red/green fluorescence intensity ratio.

### 2.13. Malondialdehyde (MDA) Assay

Intracellular MDA levels, a marker of lipid peroxidation, were measured using a Lipid Peroxidation MDA Assay Kit (Beyotime Biotechnology, Shanghai, China) according to the manufacturer’s instructions. Briefly, after the indicated treatments, cells were harvested and lysed with RIPA lysis buffer. The protein concentration of each sample was determined using a BCA protein assay kit. Equal amounts of protein were reacted with thiobarbituric acid (TBA) to form a red TBA-MDA adduct. Absorbance was recorded at 535 nm using a microplate reader (Molecular Devices, San Jose, CA, USA), and MDA concentrations were calculated from a standard curve and normalized to the total protein concentration of each sample.

### 2.14. Reduced Glutathione (GSH)/Glutathione Disulfide (GSSG) Assay

The intracellular redox state was assessed using a GSH/GSSG Ratio Assay Kit (Beyotime, Shanghai, China) according to the manufacturer’s instructions. After the indicated treatments, cells were harvested and homogenized. After centrifugation, the supernatant was collected for GSH and GSSG measurements. Total glutathione (GSH + GSSG) and GSSG levels were determined spectrophotometrically at 412 nm using a microplate reader. GSH concentrations and the GSH/GSSG ratio were calculated from a standard curve and normalized to the total protein concentration.

### 2.15. Intracellular Ferrous Iron (Fe^2+^) Assay

Intracellular Fe^2+^ levels were measured using an iron assay kit (Applygen, Beijing, China) according to the manufacturer’s instructions. After the indicated treatments, cells were harvested and homogenized in iron assay buffer. After centrifugation, the supernatant was collected and reacted with chromogen reagent, which forms a colored complex with Fe^2+^. Absorbance was recorded at 593 nm using a microplate reader, and Fe^2+^ concentrations were calculated from a standard curve and normalized to the total protein concentration of each sample.

### 2.16. Immunofluorescence Staining

After the indicated treatments, hPDLSCs were fixed with 4% paraformaldehyde at room temperature for 20 min and washed with PBS. Cells were permeabilized with Enhanced Immunostaining Permeabilization Buffer (Beyotime, Shanghai, China) for 20 min and blocked with 5% normal goat serum (Boster, Wuhan, China) for 30 min at room temperature. Cells were then incubated overnight at 4 °C with primary antibodies against SCD1 (1:200, cat. No. 28678-1-AP; Proteintech, Wuhan, China) and SREBP1 (1:200, 14088-1-AP; Proteintech).

After three washes with PBS, cells were incubated with FITC-conjugated AffiniPure Goat Anti-Rabbit IgG (H+L) secondary antibody (1:50; Boster, Wuhan, China) for 2 h at room temperature in the dark. Nuclei were counterstained with 4’,6-diamidino-2-phenylindole (DAPI; Boster, Wuhan, China) for 10 min. Fluorescence images were acquired using an FV3000 confocal laser scanning microscope (Olympus, Tokyo, Japan), and the mean fluorescence intensity was quantified using ImageJ v1.53t software (National Institutes of Health, Bethesda, MD, USA).

### 2.17. Cell Transfection

At 60–70% confluence, hPDLSCs were transfected with siRNA targeting *SCD* (si-SCD) or negative control siRNA (si-NC), or with an *SCD* overexpression plasmid (pcDNA3.1-SCD) or an empty vector control (pcDNA3.1) (all from Beijing Tsingke Biotech Co., Ltd., Tianjin, China). Transfections were performed in antibiotic-free Opti-MEM (Gibco, Waltham, MA, USA) using Lipofectamine 3000 reagent (Invitrogen, Waltham, MA, USA) according to the manufacturer’s instructions. The culture medium was replaced with complete medium 6 h after transfection.

For transfection-efficiency validation, cells were harvested 48 h after transfection for RNA and protein analyses. For functional experiments under compressive loading, transfected cells were subjected to 2.0 g/cm^2^ CF for 48 h after medium replacement and then harvested for subsequent analyses. The siRNA sequences and plasmid map are provided in Table S1 and Figure S1F, respectively.

### 2.18. RNA Extraction and Quantitative Real-Time PCR (RT–qPCR)

Total RNA was extracted from hPDLSCs using TRIzol reagent (Sparkjade, Shandong, China) according to the manufacturer’s instructions. RNA concentration and purity were assessed using a NanoDrop One microvolume spectrophotometer (Thermo Fisher Scientific, Waltham, MA, USA). A total of 500 ng of RNA per reaction was reverse-transcribed into cDNA using the SPARKscript II All-in-One RT SuperMix for qPCR (with gDNA Eraser) kit (Sparkjade, Shandong, China) according to the manufacturer’s protocol.

RT–qPCR was performed on a Bio-Rad real-time PCR system (Bio-Rad Laboratories, Hercules, CA, USA) using 2× SYBR Green qPCR Mix (Sparkjade, Shandong, China) in a 10 μL reaction volume. The thermocycling program was as follows: initial denaturation at 94 °C for 2 min, followed by 40 cycles of denaturation at 94 °C for 10 s, annealing at 55–60 °C for 20 s, and extension at 72 °C for 20 s. β-actin served as the internal reference gene. Relative mRNA expression levels were calculated using the 2^−ΔΔCt^ method. All primer sequences are provided in Table S2.

### 2.19. Western Blot Analysis

Total protein was extracted from hPDLSCs using RIPA lysis buffer (Boster, Wuhan, China) supplemented with 1 mM phenylmethylsulfonyl fluoride (PMSF) and a protease inhibitor cocktail. Protein concentrations were determined using a BCA protein assay kit (Boster, Wuhan, China) according to the manufacturer’s instructions. Equal amounts of protein (20 μg per sample) were separated by 10% SDS-PAGE (Yamei, Shanghai, China) and transferred onto polyvinylidene fluoride (PVDF) membranes (Merck Millipore, Burlington, MA, USA).

Membranes were blocked with 5% (*w*/*v*) non-fat dry milk (Beyotime, Shanghai, China) in Tris-buffered saline with Tween 20 (TBST) for 2 h at room temperature and then incubated overnight at 4 °C with the following primary antibodies: anti-SCD1 (1:2000, cat. No. 28678-1-AP; Proteintech, Wuhan, China), anti-SREBP1 (1:1000, cat. No. 14088-1-AP; Proteintech, Wuhan, China), anti-α-tubulin (1:8000, cat. No. 11224-1-AP; Proteintech, Wuhan, China), anti-OPN (1:2000, cat. No. 22952-1-AP; Proteintech, Wuhan, China), and anti-RUNX2 (1:1000, cat. No. 12556; Cell Signaling Technology, Danvers, MA, USA).

After three washes with TBST, membranes were incubated with HRP-conjugated secondary antibodies, including goat anti-rabbit IgG (H+L) (1:10,000) and goat anti-mouse IgG (H+L) (1:10,000) (both from Boster, Wuhan, China), for 2 h at room temperature. Signals were developed using an enhanced chemiluminescence (ECL) reagent (Boster, Wuhan, China) and imaged on a chemiluminescence imaging system (Bio-Rad Laboratories, Hercules, CA, USA). Band intensities were quantified using ImageJ v1.53t software and normalized to α-tubulin as the loading control.

### 2.20. Statistical Analysis

All experiments were performed with at least three independent biological replicates, and quantitative data are presented as mean ± standard deviation (SD). Comparisons between two groups were analyzed using an unpaired two-tailed Student’s *t*-test. For comparisons among more than two groups, one-way analysis of variance (ANOVA) followed by Tukey’s post hoc test was applied. Statistical analyses were conducted using GraphPad Prism 10 (GraphPad Software, San Diego, CA, USA). A *p*-value < 0.05 was considered statistically significant.

## 3. Results

### 3.1. Characterization of hPDLSCs and Establishment of the CF Model

**Figure 1:**
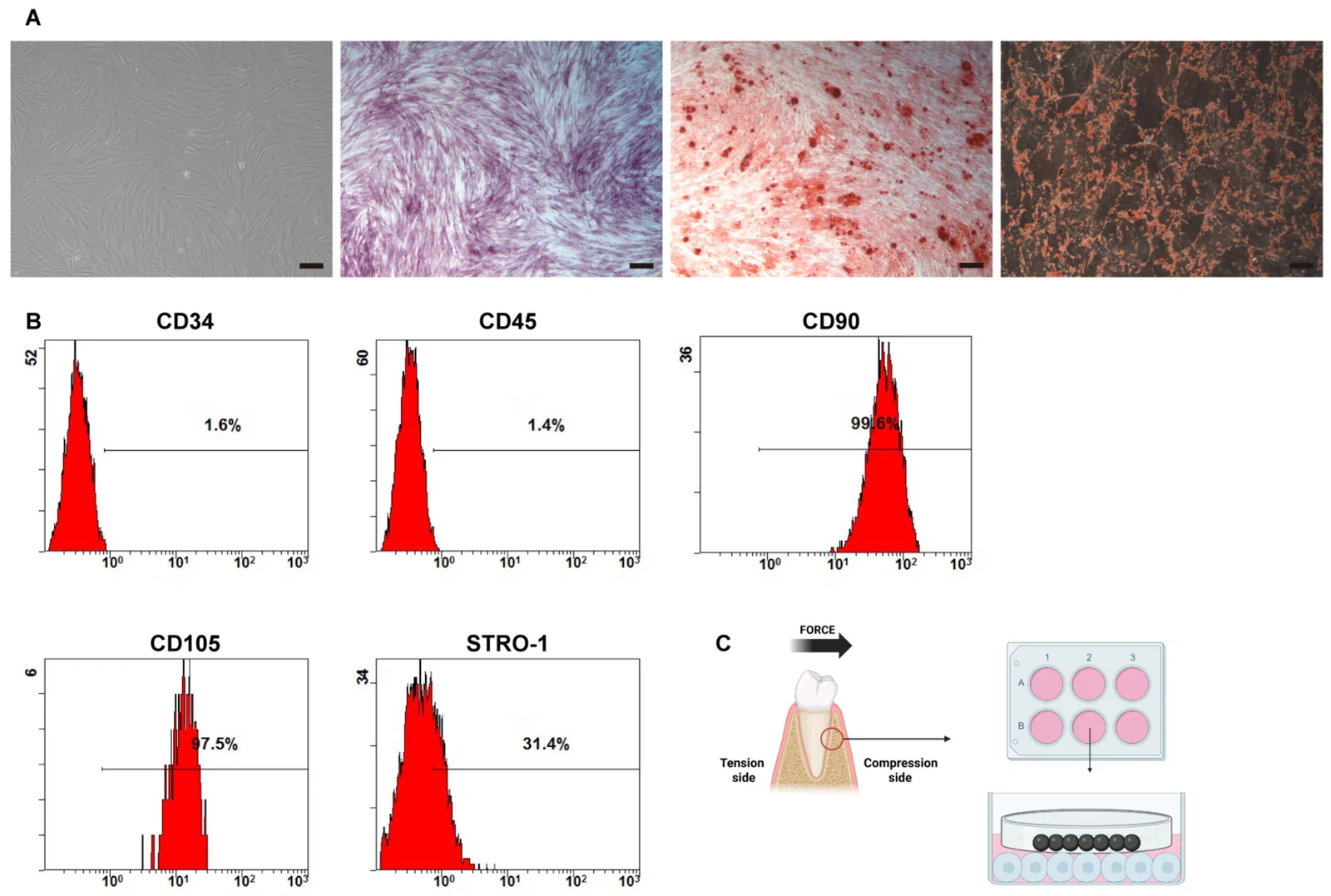
Characterization of hPDLSCs and establishment of the CF model. (**A**) Representative images showing the spindle-shaped morphology of passage-1 (P1) hPDLSCs and their osteogenic and adipogenic differentiation potential, as assessed by ALP staining, ARS staining of mineralized nodules, and Oil Red O staining of lipid droplets. Scale bars: 200 μm (morphology, ALP); 100 μm (ARS, Oil Red O). (**B**) Flow-cytometric profiles of surface markers of hPDLSCs, showing positive staining for STRO-1, CD90, and CD105 and negative staining for CD34 and CD45. (**C**) Schematic of the in vitro CF model constructed using a weight-based static loading system(A, control group; B, CF group). Created in BioRender. Tianjing, D. (2026) https://BioRender.com/h15g693, accessed on 18 September 2026.

The isolated hPDLSCs exhibited outgrowth from periodontal ligament tissue explants and displayed a characteristic elongated, spindle-shaped fibroblast-like morphology. Following osteogenic or adipogenic induction, ALP staining, ARS staining of calcified nodules, and Oil Red O staining of intracellular lipid droplets confirmed the multipotent osteogenic and adipogenic differentiation potential of hPDLSCs (Figure 1A).

Flow cytometric analysis demonstrated that the isolated cells positively expressed markers associated with mesenchymal stem cells (MSCs), including STRO-1, CD90, and CD105, while being negative for the hematopoietic lineage markers CD34 and CD45. This immunophenotypic profile is consistent with a typical MSC-like phenotype (Figure 1B).

A weight-based static compressive loading system was used to apply a vertical CF of 2.0 g/cm^2^ for 24 or 48 h. Control cells were cultured under identical conditions without compressive loading (Figure 1C).

### 3.2. RNA-Seq Analysis and Validation of SCD and SREBF1 Expression in hPDLSCs Under CF

To delineate transcriptomic responses to static compressive loading, RNA-seq was performed on hPDLSCs subjected to a 2.0 g/cm^2^ CF for 24 h, with matched unloaded cells serving as controls. The samples showed high inter-sample correlation, indicating good reproducibility of the sequencing data (Figure S1A).

**Figure 2:**
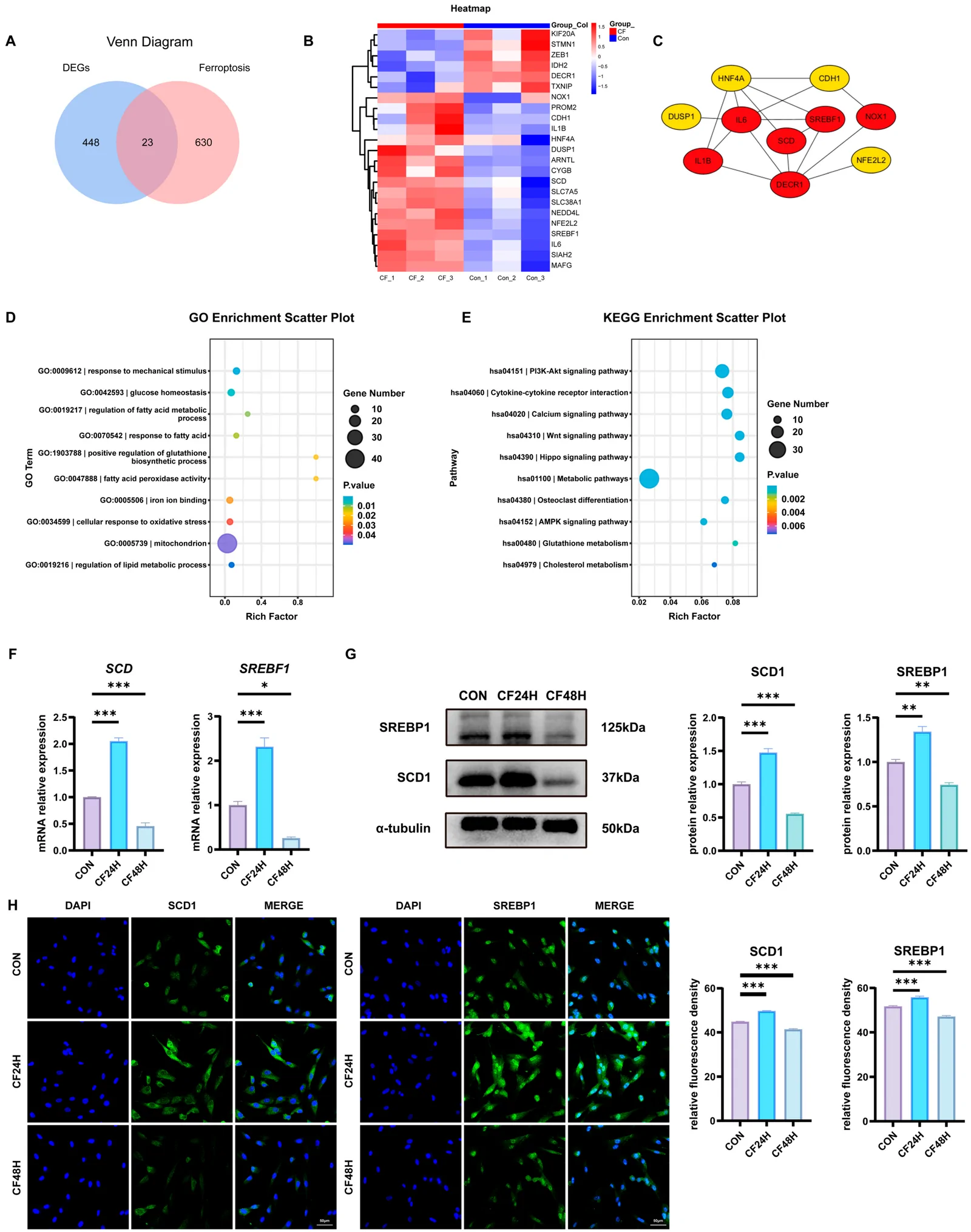
RNA-seq analysis and validation of SCD1 and SREBP1 expression in hPDLSCs under CF. (**A**) Venn diagram showing the overlap between DEGs and FRGs, identifying FRDEGs. (**B**) Heatmap of FRDEGs. (**C**) Top 10 hub genes ranked by connectivity degree in the PPI network using the cytoHubba plugin in Cytoscape. (**D**) GO enrichment analysis of DEGs. (**E**) KEGG pathway enrichment analysis of DEGs. (**F**) RT–qPCR quantification of *SCD* and *SREBF1* mRNA levels in hPDLSCs after 24 or 48 h of CF (2.0 g/cm^2^). (**G**) Representative Western blots and corresponding densitometric quantification of SCD1 and SREBP1 protein levels after 24 or 48 h of CF (2.0 g/cm^2^). Protein expression was normalized to α-tubulin and expressed relative to the control group. (**H**) Representative immunofluorescence images showing SCD1 and SREBP1 expression in hPDLSCs after 24 or 48 h of CF (2.0 g/cm^2^). Data are presented as mean ± SD (*n* = 3). * *p* < 0.05, ** *p* < 0.01, *** *p* < 0.001.

We identified a total of 653 DEGs, including 440 upregulated and 213 downregulated genes (Figure S1B,C). Among these DEGs, 23 overlapped with FRGs curated in the FerrDb database, which were designated as FRDEGs (Figure 2A). The heatmap revealed distinct expression patterns among these 23 FRDEGs, including several genes associated with lipid metabolism and redox regulation, such as *SCD*, *SREBF1*, *DECR1*, *NOX1*, *TXNIP*, and *NFE2L2* (Figure 2B). Heatmap visualization and volcano plot analysis revealed that both *SCD* and *SREBF1* were among the significantly upregulated genes at the 24 h time point (Figure 2B; Figure S1D). Consistently, PPI network analysis further supported the relevance of SREBP1 and SCD1, both of which ranked among the top 10 hub genes based on connectivity degree (Figure 2C; Figure S1E).

GO enrichment analysis showed that the DEGs were mainly associated with mitochondrial components in the cellular component category; fatty acid-related responses, lipid metabolic regulation, and oxidative stress in the biological process category; and iron ion binding in the molecular function category (Figure 2D). KEGG pathway analysis further indicated significant enrichment of metabolic pathways, particularly those related to lipid metabolism (Figure 2E). Taken together, the convergence of differential expression, PPI network connectivity, and functional enrichment related to lipid metabolism, oxidative stress, and iron-associated processes highlighted SCD1 and SREBP1 as candidate molecules for subsequent experimental validation.

We then validated the RNA-seq findings using RT–qPCR, Western blotting, and immunofluorescence staining. These assays consistently demonstrated a time-dependent biphasic response to CF: both the mRNA levels of *SCD* and *SREBF1* and protein levels of SCD1 and SREBP1 were markedly increased at 24 h but significantly decreased at 48 h compared with the corresponding unloaded controls (Figure 2F–H). These findings indicate that SCD1 and SREBP1 exhibit synchronous biphasic expression changes in response to CF.

### 3.3. CF Induces Ferroptosis in hPDLSCs and Is Attenuated by Fer-1

**Figure 3:**
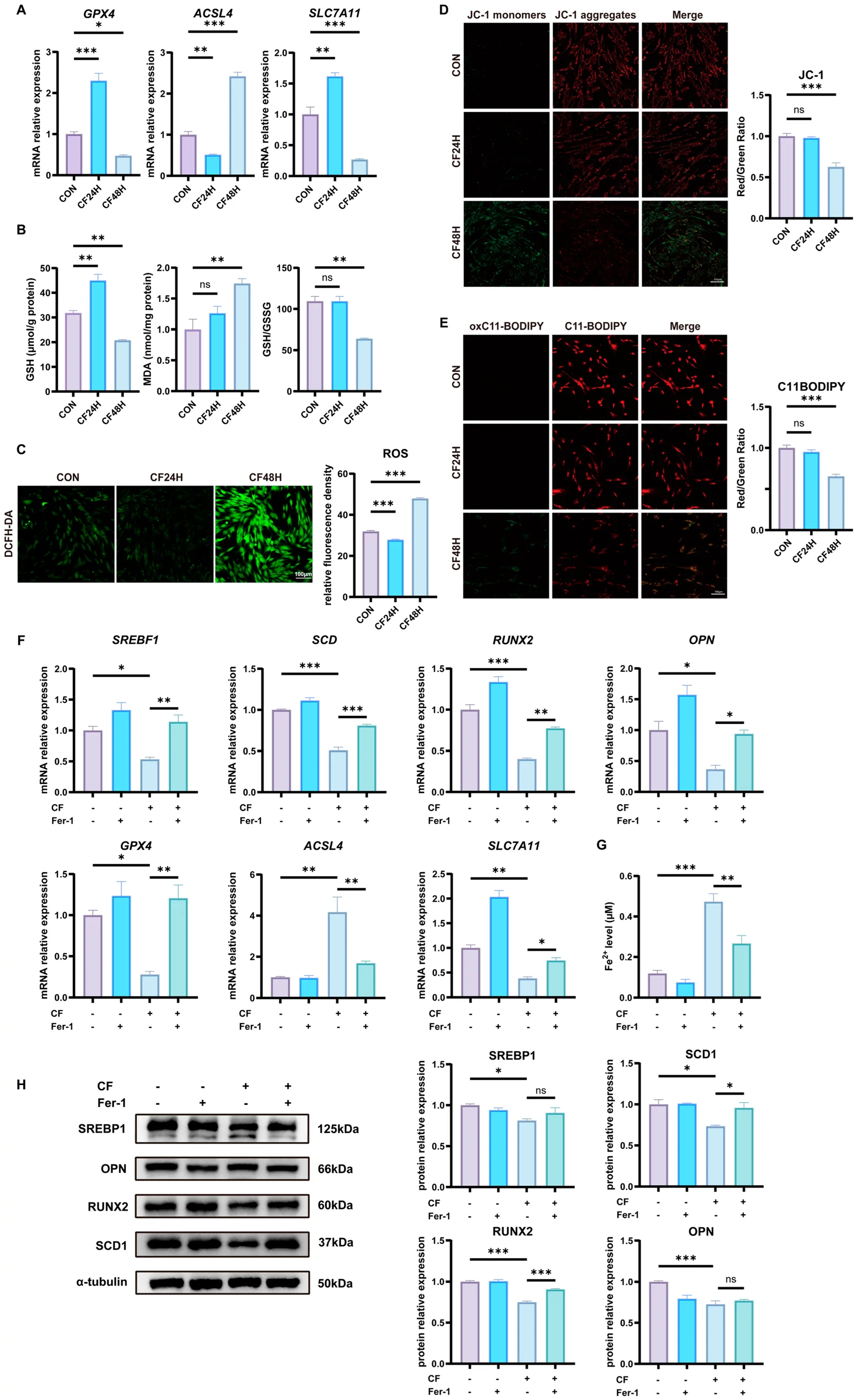
CF induces ferroptosis in hPDLSCs and is attenuated by Fer-1. (**A**) RT–qPCR analysis of *GPX4*, *ACSL4*, and *SLC7A11* mRNA expression in hPDLSCs after 24 or 48 h of CF (2.0 g/cm^2^). (**B**) Intracellular GSH and MDA levels and the GSH/GSSG ratio in hPDLSCs after 48 h of CF. (**C**) Intracellular ROS levels measured using the DCFH-DA assay. (**D**) Mitochondrial membrane potential assessed using the JC-1 assay. (**E**) Lipid ROS levels measured using C11-BODIPY 581/591 staining. (**F**) RT–qPCR analysis of *SREBP1*, *SCD*, *RUNX2*, *OPN*, *GPX4*, *ACSL4*, and *SLC7A11* mRNA expression in the CON, Fer-1, CF, and CF + Fer-1 groups. (**G**) Intracellular Fe^2+^ levels quantified using a ferrous iron assay kit. (**H**) Representative Western blots and corresponding densitometric quantification of SREBP1, SCD1, RUNX2, and OPN protein levels in the CON, Fer-1, CF, and CF + Fer-1 groups. Protein expression was normalized to α-tubulin and expressed relative to the CON group. Data are presented as mean ± SD (*n* = 3 independent experiments). * *p* < 0.05, ** *p* < 0.01, *** *p* < 0.001; ns, not significant.

After 48 h of CF, hPDLSCs exhibited a ferroptosis-associated gene expression profile, with significantly decreased mRNA levels of *GPX4* and *SLC7A11* (Solute carrier family 7 member 11) and increased mRNA expression of *ACSL4* (Acyl-CoA synthetase long-chain family member 4) (Figure 3A).

Consistently, cells exposed to CF for 48 h showed marked depletion of intracellular GSH, a decreased GSH/GSSG ratio, and elevated MDA levels, all of which are characteristic biochemical features of ferroptosis (Figure 3B). Confocal microscopy further revealed significant increases in intracellular ROS and lipid ROS levels, accompanied by a marked loss of mitochondrial membrane potential following 48 h of compression loading (Figure 3C–E).

To determine whether ferroptosis functionally contributed to these CF-induced changes, rescue experiments were performed using the specific ferroptosis inhibitor Fer-1. RT–qPCR analysis showed that, compared with cells subjected to CF alone, Fer-1 co-treatment significantly restored the mRNA expression of *SREBF1*, *SCD*, *RUNX2*, *OPN*, *GPX4*, and *SLC7A11*, whereas *ACSL4* mRNA expression was significantly reduced (Figure 3F). In parallel, CF markedly increased intracellular Fe^2+^ levels, whereas Fer-1 treatment significantly attenuated this elevation (Figure 3G). Western blotting further confirmed that Fer-1 co-treatment partially restored protein expression levels of SCD1 and RUNX2 under CF conditions (Figure 3H).

### 3.4. CF Reduces the Osteogenic Differentiation Capacity of hPDLSCs by Downregulating SCD1 Expression

**Figure 4:**
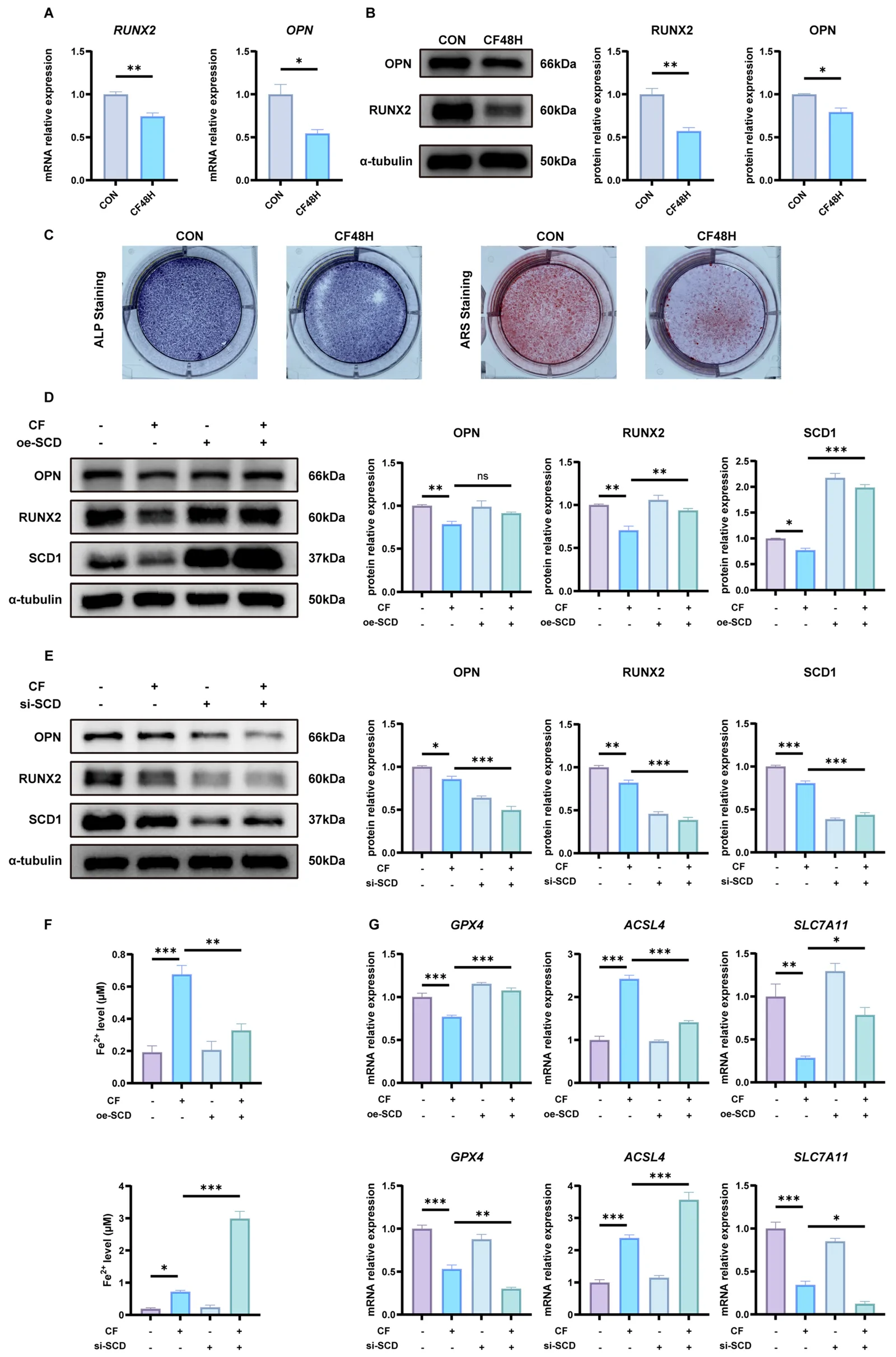
CF reduces the osteogenic differentiation capacity of hPDLSCs by downregulating SCD1 expression. (**A**) RT–qPCR analysis of *RUNX2* and *OPN* mRNA expression after 48 h of CF (2.0 g/cm^2^). (**B**) Representative Western blots and corresponding densitometric quantification of RUNX2 and OPN protein expression after 48 h of CF. Protein expression was normalized to α-tubulin and expressed relative to the control group. (**C**) ALP and ARS staining showing osteogenic differentiation capacity under CF. (**D**) Representative Western blots and corresponding densitometric quantification of SCD1, RUNX2, and OPN protein expression in the oe-NC, oe-SCD, CF + oe-NC, and CF + oe-SCD groups. (**E**) Representative Western blots and corresponding densitometric quantification of SCD1, RUNX2, and OPN protein expression in the si-NC, si-SCD, CF + si-NC, and CF + si-SCD groups. (**F**) Intracellular Fe^2+^ levels in hPDLSCs following SCD knockdown or overexpression in the presence or absence of CF. (**G**) RT–qPCR analysis of *GPX4*, *ACSL4*, and *SLC7A11* mRNA expression following SCD overexpression or knockdown in the presence or absence of CF. For panels D–G, hPDLSCs were transfected with oe-SCD, si-SCD, or the corresponding control constructs and subsequently subjected to 2.0 g/cm^2^ CF for 48 h where indicated. Data are presented as mean ± SD (*n* = 3 independent experiments). * *p* < 0.05, ** *p* < 0.01, *** *p* < 0.001; ns, not significant.

After 48 h of CF, hPDLSCs exhibited a significant decrease in the expression of the osteogenesis-related markers RUNX2 and OPN, as confirmed by RT–qPCR and Western blotting (Figure 4A,B). Consistently, ALP staining showed a reduced ALP-positive area, and ARS staining revealed decreased mineralized nodule formation, indicating that CF impairs the osteogenic differentiation capacity of hPDLSCs (Figure 4C).

To further investigate the functional role of SCD1 in this process, gain- and loss-of-function experiments were performed by overexpressing or knocking down *SCD* in hPDLSCs. Western blotting confirmed a significant increase in SCD1 protein expression following transfection with the *SCD* overexpression plasmid (Figure S1G). Compared with cells subjected to CF alone, the oe-SCD + Force group showed significant restoration of SCD1 and RUNX2. For OPN, although a trend of recovery was observed, the difference did not reach statistical significance (Figure 4D).

In parallel, *SCD* was silenced using three independent siRNAs. All three siRNA sequences effectively reduced SCD1 expression (Figure S1H). Notably, compared with cells subjected to CF alone, the si-SCD + Force group exhibited significantly lower protein expression levels of SCD1, RUNX2, and OPN (Figure 4E). To further determine whether SCD1 modulates CF-induced ferroptosis-associated alterations, intracellular Fe^2+^ levels and ferroptosis-related gene expression were examined. *SCD* knockdown markedly enhanced the CF-induced increase in intracellular Fe^2+^ levels, whereas *SCD* overexpression significantly attenuated this increase. Consistently, *SCD* overexpression partially reversed the CF-induced decreases in *GPX4* and *SLC7A11* and the increase in *ACSL4* expression, whereas *SCD* knockdown further aggravated these changes (Figure 4F,G).

### 3.5. In Vivo Downregulation of SCD1 and SREBP1 in Periodontal Tissues on the Compression Side During Orthodontic Tooth Movement

**Figure 5:**
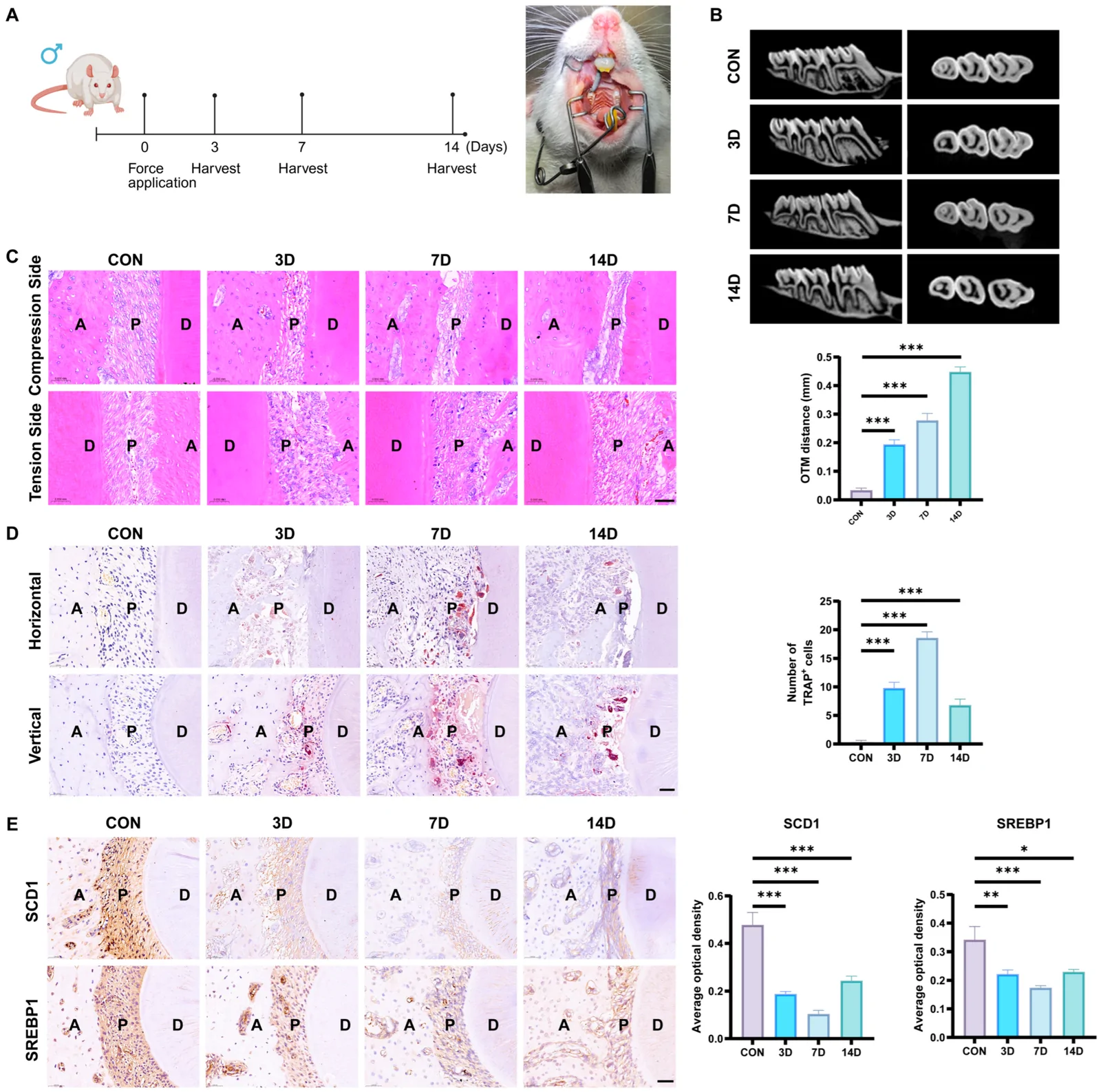
In vivo downregulation of SCD1 and SREBP1 in periodontal tissues on the compression side during OTM. (**A**) Schematic illustration of the in vivo OTM model. Created in BioRender. Tianjing, D. (2026) https://BioRender.com/de61yu0, accessed on 18 September 2026. (**B**) Representative micro-CT images of the maxilla and quantification of OTM distance. (**C**) Representative H&E staining of periodontal tissues on the compression and tension sides. (**D**) Representative TRAP staining of periodontal tissues on the compression side and quantification of TRAP-positive osteoclasts. (**E**) Representative immunohistochemical staining of SCD1 and SREBP1 in periodontal tissues on the compression side, with quantification of the positively stained area. A, alveolar bone; P, periodontal ligament; D, dentin. Data are presented as mean ± SD (*n* = 5). * *p* < 0.05, ** *p* < 0.01, *** *p* < 0.001.

To investigate the in vivo expression patterns of SCD1 and SREBP1 during alveolar bone remodeling associated with OTM, a rat OTM model was established (Figure 5A). Micro-CT imaging demonstrated progressive mesial movement of the maxillary first molar following orthodontic force application (Figure 5B). The OTM distance progressively increased from day 0 to day 14, reaching the greatest extent at day 14. Quantitative analysis showed significantly greater OTM distances in all post-force groups compared with the Control group.

H&E staining revealed characteristic histological changes: narrowing of the periodontal ligament space on the compression side and widening on the tension side, accompanied by marked tissue remodeling and cellular reorganization (Figure 5C). TRAP staining demonstrated an increased number of TRAP-positive multinucleated osteoclasts on the compression side, which peaked on day 7 and remained elevated on day 14, consistent with active bone resorption during OTM (Figure 5D).

Consistently, immunohistochemical analysis showed significantly reduced SCD1 and SREBP1 immunoreactivity in periodontal ligament tissues on the compression side at all time points examined, which is consistent with the downregulated expression pattern observed in vitro under prolonged CF (Figure 5E).

## 4. Discussion

**Figure 6:**
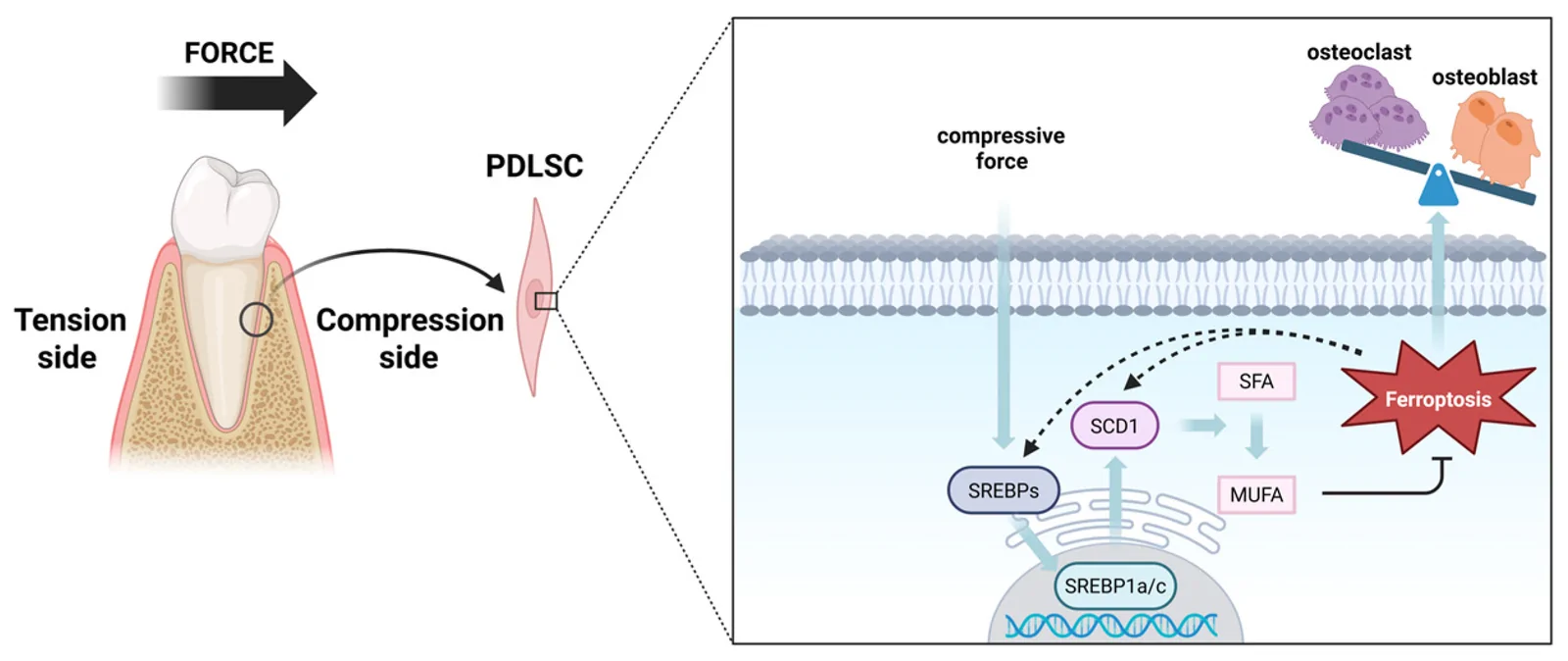
Proposed mechanism linking CF to ferroptosis and impaired osteogenic differentiation in hPDLSCs. During OTM, CF downregulates SCD1 expression in hPDLSCs (accompanied by synchronous changes in its known upstream transcription factor SREBP1), thereby promoting ferroptosis-related alterations and impairing osteogenic differentiation, which may contribute to periodontal tissue remodeling imbalance on the compression side. The dashed arrow indicates a potential reciprocal regulatory relationship between ferroptosis-associated lipid oxidative stress and lipid metabolic signaling. Created in BioRender. Tianjing, D. (2026) https://BioRender.com/au5tszi, accessed on 18 September 2026.

In this study, we identified a previously unrecognized mechanism by which prolonged CF promotes ferroptosis susceptibility and compromises the osteogenic potential of hPDLSCs during OTM through disruption of lipid metabolic homeostasis. Specifically, sustained CF downregulates SCD1 expression—accompanied by synchronous changes in its known upstream transcription factor SREBP1—resulting in increased lipid peroxidation, ferroptosis-associated cellular dysfunction, and reduced osteogenic responses (Figure 6). Functional gain- and loss-of-function experiments further demonstrated that SCD1 serves as a critical functional regulator linking mechanical stress-induced lipid metabolic dysregulation with osteogenic impairment. Collectively, our findings suggest that SCD1 downregulation may represent an important lipid metabolic response to excessive mechanical stress, contributing to ferroptosis susceptibility and suppressing the osteogenic differentiation capacity of hPDLSCs during orthodontic alveolar bone remodeling.

### 4.1. CF Induces Ferroptosis-Associated Alterations in hPDLSCs During Orthodontic Remodeling

Previous studies have shown that mechanical stimuli, including compressive or cyclic tensile forces, can induce dynamic changes in ferroptosis-related molecules in osteogenic cell lines [29]. However, whether orthodontic mechanical loading induces ferroptosis in periodontal ligament-derived stem cells and contributes to impaired bone remodeling remains largely unexplored. Consistent with previous findings, our CF model, which mimics the compression side microenvironment during OTM, revealed time-dependent alterations in ferroptosis-related and lipid metabolism-related molecules in hPDLSCs.

In two-dimensional periodontal ligament cell models, a static CF of approximately 2.0 g/cm^2^ for 24 h has been commonly used to induce reproducible mechanobiological responses while maintaining relatively high cell viability [40,41]. This loading condition therefore provides an appropriate balance between inducing a measurable cellular response and avoiding excessive compression-related injury. Accordingly, 2.0 g/cm^2^ for 24 h was selected for RNA sequencing in the present study to characterize the transcriptional response of hPDLSCs to CF. In addition, the loading period was extended to 48 h to investigate the effects of prolonged mechanical stimulation, as previous studies have demonstrated that the responses of PDLSCs to compressive loading vary with the duration of stimulation [42,43].

Both SCD1 and SREBP1 were transiently upregulated at 24 h but significantly decreased after 48 h of compression, accompanied by reciprocal changes in ferroptosis-related markers. The early elevation of SCD1 and SREBP1 may represent an adaptive metabolic response to mechanical stress, potentially enhancing lipid remodeling and maintaining membrane stability to counteract initial oxidative damage. However, prolonged compression may exceed this adaptive capacity, resulting in impaired lipid metabolic regulation and increased susceptibility to ferroptotic damage.

At the 48 h time point, hPDLSCs exhibited classical ferroptosis-associated characteristics, including decreased *GPX4* and *SLC7A11* mRNA expression, increased *ACSL4* mRNA expression, mitochondrial dysfunction, reduced GSH/GSSG ratio, elevated MDA levels, increased total ROS levels, lipid ROS, and enhanced intracellular Fe^2+^ levels [44]. These findings indicate that sustained CF drives a transition from adaptive metabolic regulation to ferroptotic damage.

Importantly, treatment with the ferroptosis inhibitor Fer-1 markedly alleviated compression-induced ferroptosis-associated alterations and restored osteogenesis-related markers, supporting a functional role of ferroptosis in compression-induced impairment of the osteogenic differentiation of hPDLSCs. Fer-1 also restored the CF-induced reduction in SREBP1 and SCD1 expression. Interestingly, in the absence of CF, Fer-1 treatment produced a modest upward trend in SREBP1 and SCD1 mRNA levels, whereas the corresponding protein levels remained relatively stable. As a potent radical-trapping antioxidant, Fer-1 suppresses lipid radical propagation and lipid peroxidation [45]. Therefore, even in the absence of pronounced ferroptotic stress, Fer-1 may modestly influence the basal lipid oxidative environment and thereby affect lipid metabolism-associated transcriptional responses, which could account for the upward trend in SREBP1 and SCD1 mRNA expression. Under CF conditions, where lipid peroxidation is markedly enhanced, the antioxidant activity of Fer-1 may additionally contribute to the recovery of SREBP1 and SCD1 expression by alleviating the disturbed lipid oxidative environment.

### 4.2. SCD1-Mediated Lipid Metabolic Regulation Contributes to Compression-Induced Suppression of Osteogenic Differentiation

Lipid metabolic status is a critical determinant of ferroptosis susceptibility, as alterations in membrane lipid composition and accumulation of peroxidation-prone lipids directly influence the extent of oxidative damage. SCD1 is a key rate-limiting fatty acid desaturase that promotes the conversion of SFAs into MUFAs, thereby maintaining lipid homeostasis and limiting lipid peroxidation. SREBP1 functions as an important transcriptional regulator of lipid metabolism and controls the expression of multiple lipogenic genes, including direct transcriptional regulation of SCD1. Although the regulatory relationship between SREBP1 and SCD1 has been well established in various cellular contexts based on previous literature [27,28], whether these lipid metabolic regulators participate in mechanical stress-induced responses during orthodontic remodeling remains unclear.

Our transcriptomic analysis further supported the involvement of ferroptosis-related metabolic alterations in hPDLSCs exposed to CF. Among the CF-responsive DEGs, 23 overlapped with ferroptosis-related genes in FerrDb. Functional enrichment analysis highlighted pathways related to lipid metabolism, oxidative stress, mitochondrial components, and iron ion binding, while PPI network analysis identified SCD1 and SREBP1 among the highly connected hub genes. Together, these findings provided the rationale for selecting SCD1 and SREBP1 for subsequent experimental validation.

In the present study, prolonged CF significantly reduced the expression of both SCD1 and SREBP1 in hPDLSCs, accompanied by enhanced lipid peroxidation, reduced antioxidant capacity, and ferroptosis-associated alterations. These findings suggest that decreased SCD1 expression under excessive mechanical stress may contribute to lipid metabolic dysregulation and increased ferroptosis susceptibility in hPDLSCs. Previous studies have shown that inhibition of SCD1 enhances lipid peroxidation and ferroptosis susceptibility in multiple cell types [46,47], supporting the potential involvement of SCD1-mediated lipid metabolic regulation in ferroptosis-associated cellular responses.

Increasing evidence from other disease models suggests that the relationship between lipid metabolic regulation and ferroptosis may not be strictly unidirectional. SREBP1-mediated lipogenic regulation and SCD1-dependent MUFA synthesis can modulate membrane lipid composition and ferroptosis susceptibility, whereas changes in pro-ferroptotic lipid composition and ferroptosis-associated oxidative states may, in turn, influence SREBP1-mediated lipogenic signaling and SCD1 expression [48,49]. Consistent with this concept, our finding that Fer-1 restored SREBP1 and SCD1 expression under CF conditions suggests that ferroptosis-associated lipid oxidative stress may, in turn, affect SREBP1- and SCD1-associated lipid metabolic regulation in mechanically stressed hPDLSCs. Moreover, the modest increase in SREBP1 and SCD1 mRNA levels following Fer-1 treatment in the absence of CF, despite limited changes at the protein level, further supports the possibility that lipid oxidative status may influence lipid metabolic signaling involving SREBP1 and SCD1. Therefore, lipid metabolic regulation involving SREBP1 and SCD1 and ferroptosis-associated lipid peroxidation may interact through a potential bidirectional regulatory network rather than a strictly linear pathway.

To determine whether SCD1 is functionally involved in compression-induced osteogenic impairment rather than merely representing a stress-associated alteration, gain- and loss-of-function experiments were performed. Under sustained compressive loading, *SCD* overexpression significantly restored RUNX2 protein expression, while OPN showed a recovery trend that did not reach statistical significance. Conversely, *SCD* knockdown further suppressed the expression of osteogenic markers. The lack of statistical significance in OPN recovery may be attributed to the relatively short duration of CF treatment (48 h) or the inherent stability of OPN protein, which may require a longer time to reflect changes at the post-transcriptional level. In contrast, RUNX2, as an upstream master transcription factor of osteogenesis, responds more rapidly to SCD1-mediated metabolic changes, consistent with its role as an early osteogenic marker.

These findings indicate that SCD1 is not merely a passive consequence of mechanical stress but actively contributes to maintaining the osteogenic differentiation capacity of hPDLSCs under mechanical loading. Together with the Fer-1 rescue results, these findings suggest that SCD1 may represent a potential regulatory link between mechanical stress-induced lipid metabolic dysregulation and suppression of osteogenic differentiation.

Previous studies have also highlighted the importance of lipid metabolic regulation in skeletal homeostasis, showing that SCD1-related lipid metabolic regulation influences MSC osteogenesis, bone repair, and fatty acid-mediated regulation of bone metabolism [50,51,52]. These findings further support the potential role of SCD1-mediated lipid homeostasis in regulating osteogenic responses during orthodontic bone remodeling.

### 4.3. In Vivo Validation and Clinical Implications for Orthodontic Bone Remodeling

To further validate the biological relevance of our in vitro findings, SCD1 and SREBP1 expression was examined in a rat OTM model. Consistent with the in vitro CF model, immunohistochemical analysis revealed a significant reduction in SCD1 and SREBP1 expression in periodontal ligament tissues on the compression side, suggesting that mechanical stress-induced lipid metabolic disturbance also occurs in the physiological orthodontic environment.

Micro-CT analysis demonstrated a progressive increase in OTM distance throughout the observation period, with the greatest displacement observed at day 14. This time-dependent tooth movement is consistent with the continuous periodontal and alveolar bone remodeling induced by sustained orthodontic force and is in agreement with previous rat OTM studies [34,35,36,37]. These findings further confirmed the successful establishment of the rat OTM model. The 3-, 7-, and 14-day observation periods were selected based on the temporal characteristics of periodontal remodeling during OTM and have been widely used in experimental rat OTM models to evaluate dynamic molecular and tissue-level responses to orthodontic force [34,35,36,37]. These time points broadly represent early, intermediate, and later remodeling responses, encompassing inflammatory activation, osteoclast recruitment, alveolar bone resorption, and continued periodontal adaptation. This temporal design allowed us to evaluate changes in SCD1 and SREBP1 expression during the progression of OTM and relate them to periodontal and alveolar bone remodeling. Unlike the transient upregulation of SCD1 and SREBP1 observed at 24 h in vitro, this adaptive phase was not detected in vivo. This discrepancy may be partly attributed to the different sampling time points: the earliest in vivo observation was performed at day 3 after OTM, when early compensatory responses may have already occurred and subsequently diminished. Moreover, the complex tissue environment involving multiple cell types—including osteoblasts, osteoclasts, fibroblasts, and immune cells—may influence the intensity and duration of these metabolic responses. Together, the consistent downregulation of SCD1 and SREBP1 across in vitro and in vivo models supports the potential involvement of SCD1-associated lipid metabolic regulation in orthodontic mechanical responses and alveolar bone remodeling.

From a clinical perspective, our findings provide a potential explanation for how excessive mechanical loading may disturb the balance of orthodontic bone remodeling. During physiological OTM, controlled bone resorption on the compression side is essential for progressive tooth movement. However, when mechanical stress exceeds the adaptive capacity of periodontal tissues, excessive lipid peroxidation and ferroptosis-associated damage may accumulate, thereby suppressing osteogenic differentiation and disrupting the delicate balance between bone resorption and bone formation.

Given the important roles of oxidative damage, lipid metabolic dysregulation, and ferroptosis in this process, targeting ferroptosis-related injury or restoring SCD1-mediated lipid homeostasis may represent a promising antioxidant therapeutic strategy for improving periodontal tissue adaptation and optimizing orthodontic bone remodeling. Such interventions could potentially reduce the risk of pathological complications such as root resorption, alveolar bone dehiscence, and fenestration during orthodontic treatment, thereby enhancing treatment safety and stability.

### 4.4. Limitations and Future Perspectives

This study expands our understanding of the biological processes underlying OTM. Nevertheless, several limitations should be acknowledged.

First, although our additional gain- and loss-of-function experiments demonstrated that SCD1 modulates CF-induced ferroptosis-associated alterations and osteogenic responses in hPDLSCs, the precise mechanisms underlying the interaction between SCD1-mediated lipid metabolic regulation and ferroptosis remain to be further elucidated. Future studies are warranted to determine whether lipid metabolic regulation involving SREBP1 and SCD1 and ferroptosis-associated lipid oxidative stress are connected through a bidirectional regulatory relationship or feedback mechanism under mechanical stress.

Second, although our in vivo experiments confirmed the downregulation of SCD1 and SREBP1 on the compression side during OTM, functional validation through in vivo SCD1 modulation or ferroptosis intervention, such as Fer-1 administration or SCD1-targeted gene therapy, was not performed. Future studies incorporating SCD1-targeted approaches or ferroptosis inhibition strategies in vivo are needed to further confirm their translational potential for orthodontic bone remodeling.

Third, it is important to note that while SREBP1 and SCD1 showed synchronous expression changes under CF, and the direct transcriptional regulation of SCD1 by SREBP1 has been well established in multiple cell types in the previous literature [27,28], we did not perform additional validation experiments (such as dual-luciferase reporter assays, ChIP, or *SREBF1* overexpression/knockdown) to confirm this regulatory relationship specifically in hPDLSCs under mechanical stress. Therefore, this study primarily focuses on the functional role of SCD1, with SREBP1 serving as an indicator of altered lipogenic signaling.

Finally, the upstream mechanotransduction pathways governing SCD1 expression in response to mechanical loading—including potential involvement of the YAP/TAZ pathway, integrin signaling, or calcium channels—require further investigation to fully elucidate the mechanistic link between mechanical force and lipid metabolic reprogramming.

## 5. Conclusions

Collectively, our findings demonstrate that prolonged CF downregulates SREBP1 and SCD1 expression—accompanied by synchronous changes in its known upstream transcription factor SREBP1—induces ferroptosis-associated lipid dysregulation and impairs the osteogenic differentiation capacity of hPDLSCs during OTM. These findings identify SCD1 as a key functional regulator linking mechanical stress to ferroptotic osteogenic impairment and suggest that targeting SCD1-mediated lipid homeostasis may represent a promising antioxidant therapeutic strategy for optimizing orthodontic bone remodeling and improving periodontal tissue adaptation.

## Data Availability

The RNA-sequencing data generated in this study have been deposited in the NCBI Gene Expression Omnibus (GEO) database under accession number GSE347200 and are available at https://www.ncbi.nlm.nih.gov/geo/query/acc.cgi?acc=GSE347200 (accessed on 18 September 2026). Other data supporting the findings of this study are included in the article and Supplementary Materials or are available from the corresponding authors upon reasonable request.

## Supplementary Materials

The following supporting information can be downloaded at https://www.mdpi.com/article/10.3390/antiox15101292/s1, Table S1. Sense and Antisense Sequences of siRNAs Targeting *SCD*; Table S2. Primer Sequences Used for RT–qPCR. Figure S1. Transcriptomic Profiling and Validation of *SCD* Overexpression and Knockdown Efficiency.

## Abbreviations

The following abbreviations are used in this manuscript:

OTM	Orthodontic tooth movement
hPDLSCs	Human periodontal ligament stem cells
ROS	Reactive oxygen species
SCD1	Stearoyl-CoA desaturase 1
SREBP1	Sterol regulatory element-binding protein 1
CF	Compressive force
GPX4	Glutathione peroxidase 4
Fer-1	Ferrostatin-1
MDA	Malondialdehyde
GSH	Glutathione
GSSG	Glutathione disulfide
SLC7A11	Solute carrier family 7 member 11
ACSL4	Acyl-CoA synthetase long-chain family member 4
