# Supplementary material for: Compressive Force During Orthodontic Tooth Movement Triggers Ferroptosis and Suppresses Osteogenic Differentiation of Human Periodontal Ligament Stem Cells Through SCD1 Downregulation
Source: Antioxidants (Basel). 2026 Oct 5;15(10):1292. doi: 10.3390/antiox15101292 (PMC13700817; doi:10.3390/antiox15101292)
Supplement: Supplementary file 1 [file antioxidants-15-01292-s001.zip › Supplementary_Materials_Revised.pdf]

**Table S1.** Sense and antisense sequences of siRNAs targeting *SCD*. (Accession number: NM_005063.5).

| siRNA | Sequence (5′–3′)(F=forward; R=reverse) | |
|---|---|---|
| siSCD-1 | GAUAUGCUGUGGUGCUUAA(dT)(dT) | UUAAGCACCACAGCAUAUC(dT)(dT) |
| siSCD-2 | CCAGAAUGAUGUCUAUGAA(dT)(dT) | UUCAUAGACAUCAUUCUGG(dT)(dT) |
| siSCD-3 | GGUUGAAUAUGUCUGGAGA(dT)(dT) | UCUCCAGACAUAUUCAACC(dT)(dT) |

**Table S2.** Primer sequences used for qRT-PCR.

| Gene | Accession number | Forward primer (5′–3′) | Reverse primer (5′–3′) |
|---|---|---|---|
| SCD | NM_005063.5 | TCGTTGCCACTTTCTTGCGATAT | TAGGGAAAGGAGTGGTGGTAGTT |
| SREBF1 | NM_004176.5 | ACAGTGACTTCCCTGGCCTAT | GCATGGACGGGTACATCTTCAA |
| GPX4 | NM_002085.5 | GAGGCAAGACCGAAGTAAACTAC | CCGAACTGGTTACACGGGAA |
| ACSL4 | NM_004458.3 | CATCCCTGGAGCAGATACTCT | TCACTTAGGATTTCCCTGGTCC |
| SLC7A11 | NM_014331.4 | TCTCCAAAGGAGGTTACCTGC | AGACTCCCCTCAGTAAAGTGAC |
| RUNX2 | NM_001024630.4 | CCCGTGGCCTTCAAGGT | CGTTACCCGCCATGACAGTA |
| OPN | NM_000582.3 | TCCTAGCCCCACAGACCCTT | CACACTATCACCTCGGCCAT |
| β-actin | NM_001101.5 | TGGCACCCAGCACAATGAA | CTAAGTCATAGTCCGCCTAGAAGCA |

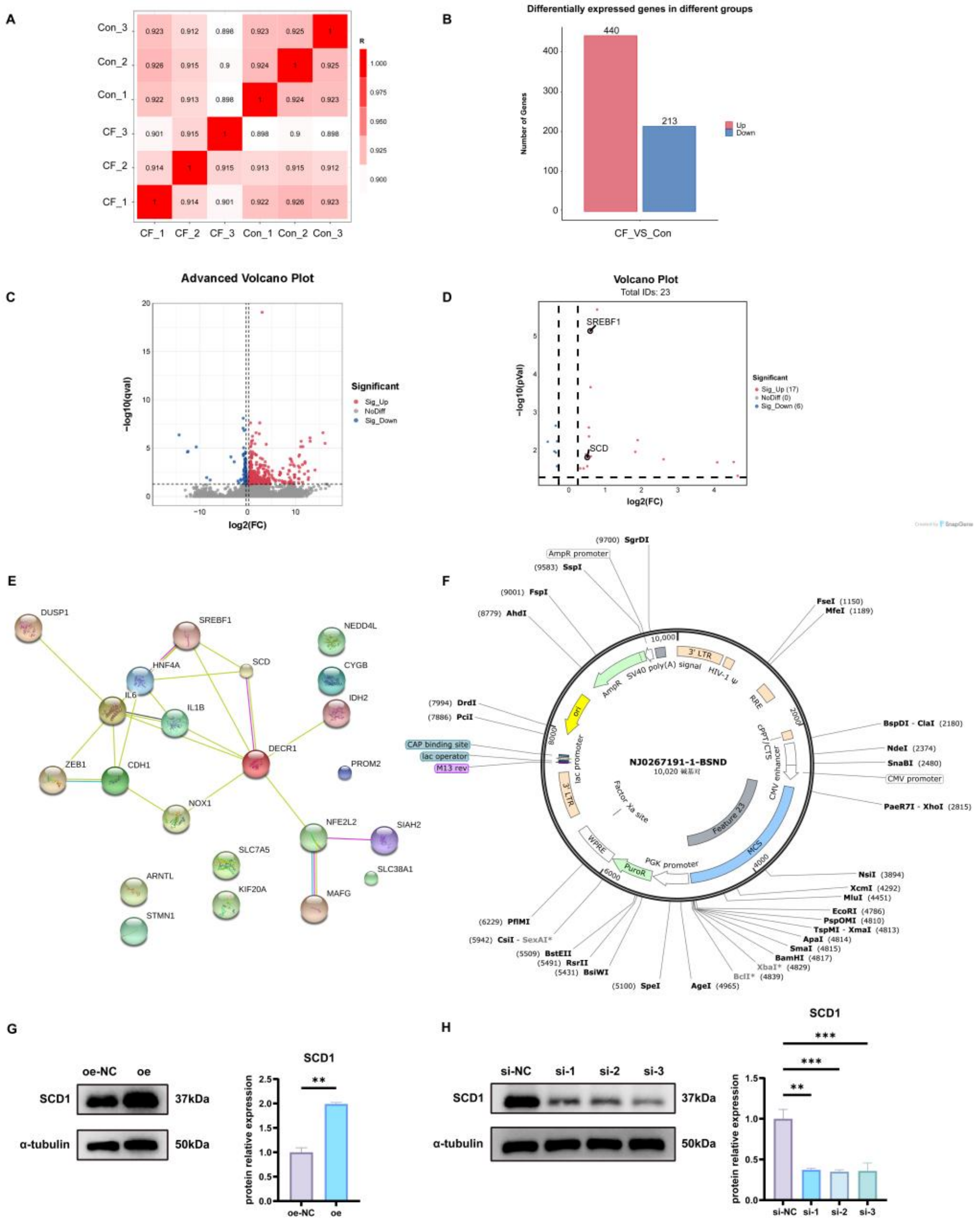


**Figure S1.** Transcriptomic profiling and validation of *SCD* overexpression/knockdown efficiency. (**A**) Sample correlation analysis of the RNA-seq dataset. (**B**) Bar chart summarizing DEGs between the CON and CF groups. (**C**) Volcano plot of DEGs. (**D**) Volcano plot of FRDEGs. (**E**) PPI network of FRDEGs generated using the STRING database. (**F**) Schematic map of the overexpression plasmid construction. (**G**) Western blot validation of *SCD* overexpression efficiency. (**H**) Western blot validation of *SCD* knockdown efficiency mediated by siRNA. Data are presented as mean ± SD (n = 3). **$p < 0.01$, ***$p < 0.001$.
